# Supplementary material for: Identification of photoperiod-induced specific miRNAs in the adrenal glands of Sunite sheep (Ovis aries)
Source: Front Vet Sci. 2022 Jul 22;9:888207. doi: 10.3389/fvets.2022.888207 (PMC9354845; doi:10.3389/fvets.2022.888207)
Supplement: Supplementary file 1 [file Data_Sheet_1.ZIP › Supplementary file/miRDeep2 file.zip!/miRDeep2 file/Know_Structure/LP42A1/oar-mir-23a.pdf]

miRBase precursor	: oar-mir-23a
Total read count	: 60378
oar-miR-23a read count	: 30244
oar-miR-23b read count	: 30132
remaining reads	: 2

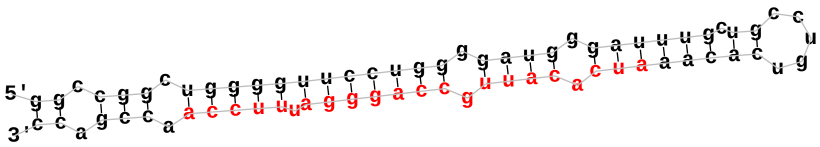

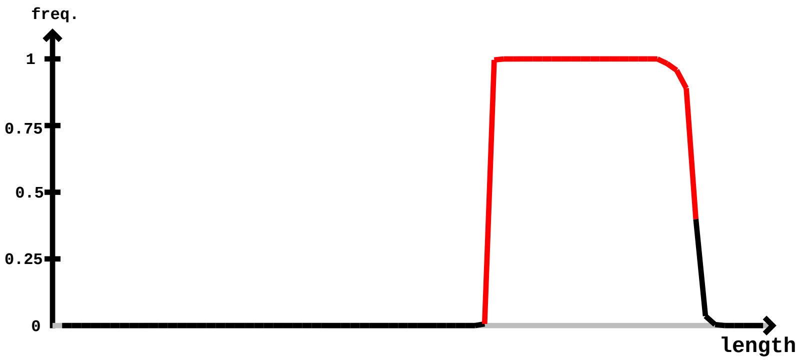


```
                                          oar-miR-23a
                                          oar-miR-23b
5'- ggccggcuggggguuccuggggaugggauuugcugccugucacaaaucacauugccagggauuuccaaccgacc   -3'    exp
    ((.(((.((((((((((((.((((.((((((.((.....)))))))).)))).))))))).))))).))).))    reads      mm         sample
    ........gggguuccuggggauggga..............................................    1          0          seq
    ........gggguuccuggggaugggauu............................................    1          0          seq
    ...........................................aaucacauugccagggau............    1          0          seq
    ...........................................aaucacauugccagggauu...........    1          0          seq
    ...........................................aaucacauugccagggauuu..........    12         0          seq
    ...........................................aaucacauugccagggauuuc.........    11         0          seq
    ...........................................aaucacauugccagggauuucc........    117        0          seq
    ...........................................aaucacauugccagggauuucca.......    40         0          seq
    ...........................................aaucacauugccagggauuuccaa......    1          0          seq
    ............................................aucacauugcUagggau............    1          1          seq
    ............................................aucacauugccagggaC............    1          1          seq
    ............................................aucacauugccagggau............    5          0          seq
    ............................................aucacauugccUgggauu...........    2          1          seq
    ............................................aucacauugccagggauu...........    511        0          seq
    ............................................aucacauugcUagggauu...........    2          1          seq
    ............................................aucacauugccagggauuu..........    747        0          seq
    ............................................aucacauugccagggauuuc.........    2016       0          seq
    ............................................aucacauugccagggauuucc........    14698      0          seq
    ............................................aucacauugccagggauuucca.......    10897      0          seq
    ............................................aucacauugccagggauuuccaa......    964        0          seq
    ............................................aucacauugccagggauuuccaac.....    98         0          seq
    ............................................aucacauugccagggauuuccaacc....    2          0          seq
    .............................................ucacauugccagggauu...........    4          0          seq
    .............................................ucacauugccagggauuu..........    1          0          seq
    .............................................ucacauugccagggauuuc.........    6          0          seq
    .............................................ucacauugccagggauuucc........    38         0          seq
    .............................................ucacauugccagggauuucca.......    45         0          seq
    .............................................ucacauugccagggauuuccaa......    3          0          seq
    .............................................ucacauugccagggauuuccaac.....    10         0          seq
    .............................................ucacauugccagggauuuccaacc....    2          0          seq
    ..............................................cacauugccagggauuucc........    1          0          seq
    ...............................................acauugccagggauuucc........    3          0          seq
    ...............................................acauugccagggauuucca.......    4          0          seq
```
